# Efficacy and Safety of Axiostat^®^ Hemostatic Dressing in Aiding Manual Compression Closure of the Femoral Arterial Access Site in Patients Undergoing Endovascular Treatments: A Preliminary Clinical Experience in Two Centers

**DOI:** 10.3390/jpm13050812

**Published:** 2023-05-11

**Authors:** Roberto Minici, Raffaele Serra, Claudio Maglia, Giuseppe Guzzardi, Marco Spinetta, Federico Fontana, Massimo Venturini, Domenico Laganà

**Affiliations:** 1 Radiology Unit, Dulbecco University Hospital, 88100 Catanzaro, Italy; miniciroberto@gmail.com (R.M.); claudiomaglia@hotmail.it (C.M.); 2 Vascular Surgery Unit, Department of Medical and Surgical Sciences, Magna Graecia University of Catanzaro, Dulbecco University Hospital, 88100 Catanzaro, Italy; rserra@unicz.it; 3 Radiology Unit, Maggiore della Carità University Hospital, 28100 Novara, Italy; giuseppe.guzzardi@maggioreosp.novara.it (G.G.); marcospinetta90@gmail.com (M.S.); 4 Diagnostic and Interventional Radiology Unit, ASST Settelaghi, Insubria University, 21100 Varese, Italy; federico.fontana@uninsubria.it (F.F.); massimo.venturini@uninsubria.it (M.V.); 5 Department of Experimental and Clinical Medicine, Magna Graecia University of Catanzaro, 88100 Catanzaro, Italy

**Keywords:** hemostasis, vascular access site, common femoral artery, femoral arterial access site, manual compression, hemostatic dressing, hemostatic pad, VASC, vascular access site complications, bleeding-related complications

## Abstract

Background: Hemostasis of the femoral arterial access site by manual compression or a vascular closure device is critical to the safe completion of any endovascular procedure. Previous investigations evaluated the hemostatic efficacy at the radial access site of some chitosan-based hemostatic pads. This study aims to assess the efficacy and safety of a new chitosan-based hemostatic dressing, namely Axiostat^®^, in aiding manual compression closure of the femoral arterial access site in patients undergoing endovascular treatments. Furthermore, the outcomes were compared with evidence on manual compression alone and vascular closure devices. Methods: This investigation is a two-center retrospective analysis of 120 consecutive patients who had undergone, from July 2022 to February 2023, manual compression closure of the femoral arterial access site aided by the Axiostat^®^ hemostatic dressing. Endovascular procedures performed with introducer sheaths ranging from 4 Fr to 8 Fr were evaluated. Results: Primary technical success was achieved in 110 (91.7%) patients, with adequate hemostasis obtained in all cases of prolonged manual compression requirements. The mean time-to-hemostasis and time-to-ambulation were 8.9 (±3.9) and 462 (±199) minutes, respectively. Clinical success was achieved in 113 (94.2%) patients, with bleeding-related complications noted in 7 (5.8%) patients. Conclusions: Manual compression aided by the Axiostat^®^ hemostatic dressing is effective and safe in achieving hemostasis of the femoral arterial access site in patients undergoing endovascular treatment with a 4–8 Fr introducer sheath.

## 1. Introduction

The common femoral artery is the primary vascular access site for many peripheral endovascular interventions. It provides easy antegrade access to the ipsilateral lower extremity and retrograde access to the remaining arterial tree, thus enabling numerous endovascular procedures aimed at restoring vessel caliber, removing a thrombus, occluding a vessel, retrieving foreign bodies, or delivering drugs or medical devices. Arterial introducer sheaths ranging in size from 4 to 8 Fr are used in most interventional radiology procedures [1,2,3,4,5,6,7,8,9,10,11,12]. After the removal of the introducer sheath, achieving hemostasis of the vascular access site by manual compression (MC) or a vascular closure device (VCD) is critical to the safe completion of any endovascular procedure [13]. Although time-to-ambulation (TTA) and time-to-hemostasis (TTH) are significantly shortened by the use of VCDs, the incidence of vascular access site complications (VASCs) remains between 5% and 12% [11,13,14,15,16,17,18,19,20,21,22]. Furthermore, the technical failure of VCDs is around 1.1–8.7% [11,14,15,16,17,21,23]. For the above reasons, except in one-day procedures, the use of VCDs is still controversial, and hemostasis by manual compression still has a key role in the case of introducers not exceeding 8 Fr in caliber with an underlying bone structure to adequately compress the access site [3,24]. Axiostat^®^ (Axio Biosolutions Private Ltd., Gujarat, India) is a new chitosan-based hemostatic dressing designed to promote hemostasis in bleeding wounds [25,26,27]. The biopolymeric mucopolysaccharide called chitosan was extracted and isolated from the exoskeleton of crustaceans [28]. The surface of the platelets is negatively charged and is attracted by the positive charge of the chitosan, thus encouraging the formation of the clot when the chitosan comes into contact with the blood [29,30]. Previously, the hemostatic efficacy at the arterial vascular access site of other chitosan-based hemostatic pads as an adjunct to mechanical compression was evaluated [31,32,33,34,35,36]. However, no data are available on Axiostat^®^.

This study aims to assess the efficacy and safety of the Axiostat^®^ hemostatic dressing in aiding manual compression hemostasis of the femoral arterial access site in patients undergoing endovascular treatments.

## 2. Materials and Methods

### 2.1. Study Design

This study is a two-center (Pugliese-Ciaccio Hospital, Italy–Mater-Domini University Hospital, Italy) analysis of retrospectively collected data from consecutive patients who had undergone, from July 2022 to February 2023, manual compression closure of the femoral arterial access site aided by the Axiostat^®^ hemostatic dressing. Inclusion criteria were (I) endovascular treatment with introducer sheath placement in the common femoral artery (CFA); (II) introducer sheath size ranging from 4 Fr to 8 Fr; (III) age of at least 18 years; and (IV) interventional radiologist’s decision to perform manual compression hemostasis of the vascular access site. The exclusion criteria were (I) age under 18 years; (II) prior surgery in the access area and/or groin scar; (III) previous endovascular treatments or vascular accesses or VCD use at the vascular access site; (IV); bleeding risk exceeding recommendations of the CIRSE Standards of Practice on Peri-Operative Anticoagulation Management [37]; and (V) pregnancy.

### 2.2. Treatment

As per our institution’s protocol, placement of the introducer sheath in the CFA is fluoroscopically assessed, and pressure parameters are recorded at the end of the endovascular procedure, before the introducer sheath removal.

**Figure 1:**
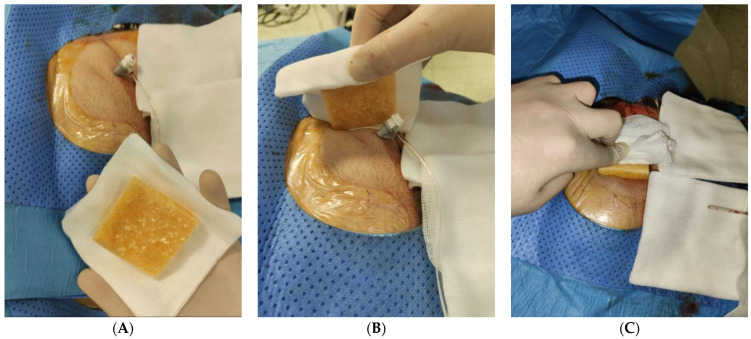
Preparation and application of the Axiostat^®^ hemostatic dressing. The skin around the vascular access site and the operators’ gloves are cleaned; Axiostat^®^ is then prepared with cotton gauze (**A**). The interventionist moves Axiostat toward the introducer sheath (**B**), removes it and performs manual compression by placing the Axiostat^®^ hemostatic dressing between their hand and the vascular access site (**C**). Upon removal of the introducer sheath, a few drops of blood spontaneously flow out of the access site, thus imbibing Axiostat^®^ with blood and triggering a charge-based adhesion between the positively charged chitosan and the negatively charged blood.

The skin around the vascular access site and the operators’ gloves are cleaned. The interventionist applies proximal pressure at the access site, removes the introducer sheath with the other hand, modulates proximal pressure to check for bleeding from the access site and then initiates manual double-hand compression with a standard technique [38]. The technique of hemostasis of the vascular access site by manual compression is in no way modified with respect to what is defined by our institutions’ protocol; the Axiostat^®^ hemostatic pad is just applied on the skin access site instead of cotton gauze (Figure 1).

Manual compression is performed for 7 min in cases of 4–6 Fr sheath sizes and for 10 min in cases of 7–8 Fr sheath sizes. At the end of these time windows, the operator inspects the access site. If an expanding hematoma or significant bleeding via the puncture site are noted, manual compression is continued until adequate hemostasis is achieved. Adequate hemostasis was defined by Schulz-Schüpke et al. [21]. Thereafter, a pressure dressing is applied to the access site and maintained for 6 h in cases of 4–6 Fr sheath size and 12 h in cases of 7–8 Fr sheath size, as per a local protocol. The pressure dressing is then removed, the vascular access site is inspected, and the patient’s ambulation is permitted if there is no evidence of vascular access site complications. The Axiostat^®^ hemostatic pad is removed by irrigating with saline and gently peeling it off. Color-coded duplex sonography is performed immediately before hospital discharge, and patients are interviewed via a telephone call after 30 days to rule out any VASCs. If any access site complaints are reported, the patient is invited for further clinical and duplex sonographic in-hospital follow-up, as per our institutions’ protocol.

### 2.3. Outcomes and Definitions

The primary objective of this study was to evaluate the efficacy and safety of the Axiostat^®^ hemostatic dressing in aiding manual compression closure of the femoral arterial access site in patients undergoing endovascular treatments. The primary efficacy endpoint was the primary technical success rate achieved by manual compression aided by the Axiostat^®^ hemostatic dressing. The primary safety endpoint was the clinical success rate. Secondary endpoints included the secondary technical success rate, the TTH, the TTA and the VASC rate.

The secondary objective of the study was to evaluate and compare the efficacy and safety between two subgroups (Group 1: sheath size 4–6 Fr; Group 2: sheath size 7–8 Fr).

The tertiary objective was to evaluate predictors of bleeding-related VASCs through logistic regression analyses.

Primary technical success was defined by adequate hemostasis achieved by 7 min (in cases of 4–6 Fr sheath sizes) or 10 min (in cases of 7–8 Fr sheath sizes) of manual compression aided by the Axiostat^®^ hemostatic dressing. Secondary technical success was defined as adequate hemostasis after the above scheme or after an additional period of Axiostat^®^-aided manual compression. Clinical success was defined by the absence of bleeding-related VASCs at follow-up. Complications were graded according to SVS reporting standards and the CIRSE classification system [39,40]. The hematoma was defined as a palpable mass with a dimension of at least 5 cm, confirmed by ultrasonography. Diagnoses of pseudoaneurysm, arteriovenous fistula, thrombosis and dissection require iconographic confirmation by imaging examination. The diagnosis of neuropathy requires specialized neurological consultation. Access site-related major bleeding is defined as a reduction in hemoglobin of at least 3 g/dL with evident bleeding, a reduction in hemoglobin of at least 4 g/dL with or without evident bleeding, or bleeding requiring blood transfusion, similar to the REPLACE-2 trial criteria [41]. Local infection is defined as an infected skin or soft tissue lesion at the vascular puncture site that requires antibiotic treatment [41]. The vascular access at CFA is considered adequate if performed below the inferior epigastric artery and above the femoral bifurcation, as per fluoroscopic evaluation. The number of punctures was calculated by considering each time the needle was pulled out of the skin after its insertion. Coagulopathy was defined as in Loffroy et al. [42]: INR greater than 1.5, partial thromboplastin time longer than 45 s, or platelet count less than 80,000/mm^3^. Adequate hemostasis is defined as no bleeding or only light superficial bleeding and no expanding hematoma, as in Schulz-Schüpke et al. [21]. Time-to-hemostasis is defined as the time elapsed (in minutes) between the introducer sheath removal and the observation of adequate hemostasis. Time-to-ambulation is defined as the time elapsed (in minutes) between applying the pressure dressing and allowing the patient to ambulate.

### 2.4. Statistical Analysis

Data were maintained in an Excel spreadsheet (Microsoft Inc., Redmond, WA, USA), and the statistical analyses were performed using SPSS software (SPSS, version 26 for Windows; SPSS Inc., Chicago, IL, USA) and R/R Studio software. Statistical analysis was based on the Modified Intention-To-Treat (MITT) population, defined as all the patients recruited who answered the questionnaire 1 day after intervention, answered the telephone interview 30 days after the procedure, underwent any clinical and ultrasound examination if required and were still alive. Additionally, included in the MITT population are patients who experienced a VASC and died or who experienced a VASC and did not complete a 30-day follow-up. The Kolmogorov–Smirnov test and the Shapiro–Wilk test were used to verify the normality assumption of the data. Data are presented as previously described [43,44,45,46]. Descriptive statistical methodologies were applied in the study. Subgroup analyses were performed. The unpaired Student t-test was used to assess statistical differences for continuous, normally distributed data. The Mann–Whitney test was used to assess statistical differences for continuous, not normally distributed data. Categorical data were assessed using the Chi-squared test or the Fisher exact test, as appropriate. Simple and multiple logistic regression analyses were performed to assess possible factors that would predict the occurrence of bleeding-related VASCs (80% of the dataset was used as a training set and the remaining 20% as a testing set). A simple logistic regression was conducted to predict the probability of class membership based on a single predictor variable. Subsequently, a multiple logistic regression was conducted to predict the probability of class membership based on multiple predictor variables. Lastly, the worst predictors were dropped from the model to avoid overfitting. Since they were low in number, they were manually selected, and then a multiple logistic regression was performed again with the remaining predictors, as previously described [47]. A *p*-value of <0.05 was considered statistically significant for the aforementioned tests.

## 3. Results

### 3.1. Study Population

During the study interval (July 2022–February 2023), 120 patients underwent manual compression closure of the femoral arterial access site aided by the Axiostat^®^ hemostatic dressing. The mean age was 68.3 years, and 50.8% of the patients were female. The mean value of the Body Mass Index was 24.7 (±5.4). The mean INR was 1.2 (±0.2) and 12 (10%) patients were affected by coagulopathy, 42 (35%) patients were on antiplatelet therapy, 34 (28.3%) were on anticoagulant therapy and 70 (58.3%) were on antiplatelet or anticoagulant therapy.

Population data are reported in Table 1.

### 3.2. Procedure Data

The most used size of vascular introducer sheath was the 5 Fr (46 patients, 38.3%), with a mean number of vascular access-site punctures per patient of 1.3 (±0.8). Eight (6.7%) patients received a thrombolytic agent during endovascular treatment. Seven patients received recombinant tissue plasminogen activator as concomitant drug therapy with endovascular treatment for acute ischemic stroke, while one patient received catheter-directed thrombolysis by intra-arterial infusion of urokinase for peripheral arterial thrombus. The mean systolic pressure before the sheath removal was 134.4 (±26.3) mmHg.

Procedure data are detailed in Table 2.

### 3.3. Outcomes

Primary technical success was achieved in 110 (91.7%) patients, with adequate hemostasis obtained in all cases of prolonged MC requirement. The mean TTH and TTA were 8.9 (±3.9) and 462 (±199) minutes, respectively. Clinical success was achieved in 113 (94.2%) patients, with bleeding-related VASCs noted in 7 (5.8%) patients.

The rate of vascular access site complications (VASCs) was 8.3%, related to two (1.7%) cases of hematoma, five (4.1%) pseudoaneurysms and three (2.5%) cases of dissection. No major bleeding events occurred. According to the CIRSE Classification System for Complications, only grade 2 or 3 events occurred. According to SVS reporting standards, no major (grade 3) VASCs were noted. Only one case of pseudoaneurysm required an invasive procedure (i.e., US-guided percutaneous thrombin injection). Medical treatment consisted of prolonged application of pressure dressing in two cases (1.7%), ultrasound-guided compression of pseudoaneurysms in four cases (3.3%), and administration of antiplatelet therapy to prevent complications of dissections in three cases (2.5%).

Details are given in Table 3.

### 3.4. Sheath Size 4–6 Fr vs. Sheath Size 7–8 Fr

A comparison of data between patients undergoing endovascular treatment with introducer sheath sizes ranging from 4 Fr to 6 Fr (Group 1) and patients undergoing endovascular treatment with introducer sheath sizes ranging from 7 Fr to 8 Fr (Group 2) has been performed. Interestingly, Group 2 (sheath size 7–8 French) consists almost exclusively of neurovascular procedures, such as carotid artery stenting and mechanical thrombectomy in ischemic stroke. Instead, Group 1 (sheath size 4–6 French) consisted of patients undergoing non-neurovascular procedures such as bleeding embolizations, chemoembolizations and percutaneous treatments of peripheral arterial disease. Therefore, the difference in sheath size exactly corresponds to the difference in procedures performed.

No statistically significant differences were observed between the group undergoing endovascular treatment with introducer sheath sizes ranging from 4 Fr to 6 Fr and the group undergoing endovascular treatment with introducer sheath sizes ranging from 7 Fr to 8 Fr, in terms of age (69.2 vs. 65.5 years; *p* = 0.317), coagulopathy (11 vs. 1 patients; *p* = 0.291), INR (1.2 vs. 1.2; *p* = 0.937), antiplatelet or anticoagulant therapy (51 vs. 19 patients; *p* = 0.343), systolic pressure ≥ 180 mmHg (10 vs. 6 patients; *p* = 0.201), VASCs (9 vs. 1; *p* = 0.450), and bleeding-related VASCs (6 vs. 1; *p* = 1).

The number of patients receiving an intraoperative thrombolytic agent was significantly higher in the 7–8 Fr group (25% vs. 1.1%; *p* < 0.001). The primary technical success rate was significantly higher in the 4–6 Fr group (97.8% vs. 71.4%; *p* < 0.001).

Details are given in Table 4.

### 3.5. Predictors of Bleeding-Related VASCs

Simple logistic regression analyses showed that coagulopathy (*p* = 0.036) and systolic pressure (*p* = 0.007) were significant single predictors of bleeding-related VASC occurrence. Multiple logistic regression models failed to find significantly reliable predictors if multiple predictor variables were considered, except for coagulopathy (*p* = 0.029). Despite the absence of statistical significance, it is noted that the group with coagulopathy is associated with an average increase of 4.55 in the log odds of bleeding-related VASC occurrence.

Details are given in Table 5.

## 4. Discussion

Hemostasis of the femoral arterial access site is critical to the safe completion of any endovascular treatment. If arterial introducers ranging in size from 4 to 8 Fr are used, hemostasis can be achieved by MC or a VCD. Both hemostasis techniques have some disadvantages that should be recognized.

MC is often time-consuming [18,48], an element that assumes particular importance in night procedures and in conditions of staff shortage and overlapping between several urgent procedures. From the patient’s perspective, MC is often significantly painful with a prolonged TTH and requires long bed rest afterwards with delayed TTA and time-to-discharge (TTD) [13,18,49]. Furthermore, during the period of forced bed rest, the patient often complains of discomfort due to back pain, an inconvenient diet, dysuria and difficult defecation [50,51].

Several investigators advocated the utility of VCDs, as shortened TTH, TTA and TTD are noted [13,14,15,16,17,18]. Increased [52], or at least not decreased [48], VASCs have been observed with the use of first-generation VCDs. A comparable VASC rate between MC and new-generation VCDs, such as the Angio-Seal VIP, and improved patient-reported outcomes with VCDs have been described in more recent reports [13,18,48]. However, VCDs still have a VASC rate of around 5 to 12% [11,13,18,19,20,21,22]. Moreover, the failure rate of VCDs in achieving access-site hemostasis is around 1.1–8.7% [11,14,15,16,17,21,23].

Hence, the use of VCDs is supported in same-day procedures since they guarantee faster hemostasis, ambulation and discharge, as well as reduced costs [3,53,54]. However, there are no guidelines recommending the use of VCDs over the MC [3] and in current clinical practice, the choice of hemostasis technique depends on the protocol, availability and experience of the local institution. The Axiostat® hemostatic dressing is not intended as a VCD, as it is a hemostatic pad that facilitates hemostasis by manual compression of the vascular access site. Therefore, it is crucial to compare the outcomes of femoral access site hemostasis using Axiostat-aided manual compression with literature data on manual compression alone and VCDs. In our investigation, the Axiostat^®^ hemostatic dressing appeared to improve the efficacy and safety of MC for femoral arterial access site hemostasis in patients undergoing endovascular treatments.

The primary technical success rate was approximately 91.7%. Thus, the failure rate of the rapid hemostasis protocol using MC and Axiostat used in our institutions (7 min for sheath sizes 4–6 Fr and 10 min for sheath sizes 7–8 Fr) was approximately 8.3%. These results are not very dissimilar from the failure rates of VCDs reported in the current literature (1.1–8.7%) [11,14,15,16,17,21,23]. Furthermore, it is worth emphasizing that both in the event of a VCD failure and in the event of the failure of our rapid hemostasis protocol, a continuation of hemostasis by MC would be required. In our experience, the secondary technical success rate was 100%, meaning that effective hemostasis was achieved in all cases, even when prolongation of hemostasis was necessary.

Gewalt et al. recorded a significantly shorter time to hemostasis in women assigned to a VCD compared with those assigned to MC (1 min [IQR, 0.5–2.0] versus 11 min [IQR, 10–15]; *p* < 0.001); a 1:1 ratio of extravascular and intravascular VCDs was adopted [19]. Similarly, Schulz-Schüpke et al. also observed a shorter time to hemostasis in patients assigned to receive VCD compared with those assigned to receive manual compression (1 min [IQR, 0.5–2.0], versus 10 min [IQR, 10–15]; *p* < 0.001). Jakobsen et al. noted a median TTH of 4 min (IQR, 3–5) in 432 patients undergoing vascular access site hemostasis with the extravascular VCD MynxGrip^®^ [55]. A recent meta-analysis by Pang et al. confirmed the advantages of TTH and TTA of the VCDs over the MC [18]. Interestingly, we observed reduced TTH with Axiostat^®^-aided MC compared to MC alone, as assessed by other reports. Other chitosan-based hemostatic pads were previously evaluated for radial artery hemostasis [31,32,33,34,35], with a lack of investigations on femoral artery hemostasis.

Postoperative hemostasis maintenance can be indirectly assessed by bleeding-related VASCs, which can serve as surrogate markers [18]. In our investigation, bleeding-related VASCs were observed in seven (5.8%) cases and VASCs in 10 (8.3%) cases, in keeping with the current literature [56,57]. Therefore, manual compression closure of the femoral arterial access site aided by the Axiostat^®^ hemostatic dressing proved to be safe in patients undergoing endovascular treatment. A current meta-analysis limited to the subgroup of randomized controlled trials highlighted a VASC rate of 8.8% for MC and 7.3% for VCD [18]. 6.7% of bleeding-related VASCs in the VCD group and 8.5% of bleeding-related VASCs in the MC group were noted by Schulz-Schüpke et al. [21]. Gewalt et al. observed 8.5% bleeding-related VASCs in the VCD group and 10.7% bleeding-related VASCs in the MC group [19]. Sharma et al. observed 5% of bleeding-related events in the VCD group and no cases in the MC group [19]. No statistically significant differences were observed in terms of bleeding-related VASCs between the VCD group and the MC group in a recent meta-analysis (4.0% vs. 5.2%—RR: 0.77, CI, 0.60–1.00, *p* = 0.05) [18]. An interesting speculation is that VCD failure increased the incidence of minor bleeding complications and partially offset the benefits of VCD on hemostasis [18,58,59,60]. In our retrospective evaluation, we did not observe major vascular complications, with only one case of pseudoaneurysm treated by US-guided percutaneous injection of thrombin. It is worth noting that most VASCs can be effectively treated by percutaneous treatment [61].

Coagulopathy and systolic pressure recorded before sheath removal were found to be single independent predictors of the onset of bleeding-related VASCs. This finding suggests that more caution should be exercised in hemostasis at the vascular access site. It could be speculated that possible remedies could include blood pressure correction before hemostasis, more prolonged hemostasis, a tighter pressure dressing or VCD hemostasis if available in the Catheterization Lab. Subgroup analysis revealed a significant reduction in the primary technical success rate in the subgroup of patients undergoing endovascular treatment with a 7–8 Fr introducer sheath. It is useful to note how this subgroup shows a significantly greater use of intraoperative thrombolytic agents and a trend, albeit not statistically significant, towards a higher systolic blood pressure and a higher percentage of patients undergoing antiplatelet or anticoagulant therapy. This figure is a consequence of the different endovascular interventions performed in the two subgroups; in the 7–8 Fr introducer subgroup, most of the operations performed were carotid artery stenting and mechanical thrombectomy in ischemic stroke.

The lack of randomization, the absence of a control group and the small population size are the main limitations of the investigation. In addition, other drawbacks of the study should be recognized. First, it is a preliminary clinical experience where the Axiostat^®^ hemostatic dressing was simply used in place of cotton gauze in our institution’s manual compression hemostasis protocol. From the first limitation derives a second, consisting of the high heterogeneity of treatments and introducer sheaths used. Third, the relative influence of periprocedural anticoagulant therapy on the time to hemostasis is difficult to interpret, thus introducing a possible confounding factor. The heterogeneity of endovascular treatments makes this confounding factor even more difficult to eliminate. Furthermore, it is worth noting that a previous meta-analysis on VCD hemostasis highlighted a significant heterogeneity in outcomes according to whether patients underwent diagnostic or therapeutic procedures, possibly due to the variability in the use of periprocedural anticoagulant therapy [62]. Fourth, the retrospectivity of the analysis provides an inferior level of evidence. Fifth, the assessment of the congruence and consistency of our data is limited by the scarcity of data in the literature.

## 5. Conclusions

The results of the current retrospective investigation demonstrate that manual compression aided by the Axiostat^®^ hemostatic dressing is effective and safe in achieving hemostasis of the femoral arterial access site in patients undergoing endovascular treatment with a 4–8 Fr introducer sheath. The absence of a control group limits the strength of our investigation. Larger, prospective, randomized, controlled trials are desirable to confirm these preliminary data and compare the efficacy and safety profiles of MC aided by Axiostat^®^, MC alone and VCDs.

Coagulopathy and systolic pressure recorded before sheath removal are reliable single independent predictors of the onset of bleeding-related VASCs.

## Figures and Tables

**Table 1:** Population data.

Variables	All Patients (*n* = 120)
Age (years)	68.3 (±14)
Sex (M/F)	59 (49.2%)/61 (50.8%)
BMI	24.7 (±5.4)
INR	1.2 (±0.2)
aPTT (s)	35.8 (±4.2)
PLT (×10^3^/µL)	404.6 (±32)
Coagulopathy	12 (10%)
Hypercoagulable state	8 (6.7%)
Diabetes mellitus	71 (59.2%)
Coronary artery disease	47 (39.2%)
Congestive heart failure	31 (25.8%)
Cerebrovascular disease	9 (7.5%)
Smoking history	65 (54.2%)
Current smoker	39 (32.5%)
Hypertension	61 (50.8%)
Hyperlipidaemia	80 (66.7%)
Chronic renal insufficiency (eGFR < 60 mL/min)	23 (19.2%)
Antiplatelet therapy	42 (35%)
Anticoagulant therapy	34 (28.3%)
Antiplatelet OR Anticoagulant therapy	70 (58.3%)
Antiplatelet AND Anticoagulant therapy	3 (2.5%)

**Table 2:** Procedure data.

Variables	All Patients (*n* = 120)
Introducer sheath size	
- 4F	19 (15.8%)
- 5F	46 (38.3%)
- 6F	27 (22.5%)
- 7F	15 (12.5%)
- 8F	13 (10.8%)
Number of punctures of vascular access-site	1.3 (±0.6)
Intraoperative Unfractionated Heparin	2000 (0–2000)
Intraoperative thrombolytic agent	8 (6.7%)
Intraoperative Antiplatelet therapy	10 (8.3%)
Systolic Pressure (mmHg)	134.4 (±26.3)
Diastolic Pressure (mmHg)	85.5 (±16.8)
Mean Arterial Pressure (mmHg)	110 (±20.6)

**Table 3:** Outcomes.

Variables		All Patients (*n* = 120)
Primary technical success		110 (91.7%)
Secondary technical success		120 (100%)
Time-to-hemostasis (min)		8.9 (±3.9)
Time-to-ambulation (min)		462 (±199)
Clinical success		113 (94.2%)
Vascular access-site complications (VASCs), no/yes		110 (91.7%)/10 (8.3%)
	Haematoma	2 (1.7%)
	Pseudoaneurysm	5 (4.1%)
	Dissection	3 (2.5%)
	AV Fistula	0 (0%)
	Major bleeding	0 (0%)
	Thrombosis	0 (0%)
	Infection	0 (0%)
	Neuropathy	0 (0%)
Bleeding-related VASCs		7 (5.8%)
SVS Grading VASCs		
	Grade 1	1 (0.8%)
	Grade 2	9 (7.5%)
	Grade 3	0 (0%)
CIRSE Grading VASCs		
	Grade 1	0 (0%)
	Grade 2	2 (1.7%)
	Grade 3	8 (6.6%)
	Grade > 3	0 (0%)
Required treatment		
	Medical	9 (7.5%)
	Percutaneous	1 (0.8%)
	Surgical	0 (0%)

**Table 4:** Comparison of data between patients undergoing endovascular treatment with introducer sheath sizes ranging from 4 Fr to 6 Fr (Group 1) and patients undergoing endovascular treatment with introducer sheath sizes ranging from 7 Fr to 8 Fr (Group 2).

Variables	Group 1 (*n* = 92) Sheath Size 4–5–6 Fr	Group 2 (*n* = 28) Sheath Size 7–8 Fr	*p* Value
Age (years)	69.2 (±13.7)	65.5 (±15.1)	0.317
Coagulopathy	11 (11.9%)	1 (3.6%)	0.291
INR	1.2 (±0.2)	1.2 (±0.2)	0.937
Intraoperative thrombolytic agent	1 (1.1%)	7 (25%)	<0.001
Antiplatelet OR Anticoagulant therapy	51 (55.4%)	19 (67.8%)	0.343
Systolic pressure ≥ 180 mmHg	10 (10.8%)	6 (21.4%)	0.201
Primary technical success	90 (97.8%)	20 (71.4%)	<0.001
VASCs	9 (9.8%)	1 (3.6%)	0.450
Bleeding-related VASCs	6 (6.5%)	1 (3.6%)	1

**Table 5:** Logistic regression analysis of predictive factors affecting bleeding-related VASC occurrence (Simple/Multiple/Multiple II).

Predictors	Coeff.	Std. Err.	Z	*p* > |z|
Coagulopathy	2.08/8.87/4.55	0.99/13.44/2.63	2.09/0.66/1.73	0.036/0.509/0.084
INR	3.33/−2.70	1.74/4.36	1.91/−0.62	0.056/0.536
Antiplatelet OR Anticoagulant therapy	0.12/−3.15	0.94/11.14	0.13/−0.28	0.999/0.777
Systolic pressure	0.08/0.35/0.12	0.03/0.36/0.06	2.68/0.99/2.18	0.007/0.321/0.029
Primary technical success	−0.82/8.89	1.17/13.61	−0.70/0.65	0.482/0.513

## Data Availability

The data presented in this study are available on request from the corresponding author. The data are not publicly available due to privacy issues.

## Abbreviations

CFA: common femoral artery; IQR: interquartile range; MC: manual compression; TTA: time-to-ambulation; TTD: time-to-discharge; TTH: Time-to-hemostasis; VASCs: vascular access site complications; VCD: vascular closure device.
